# A Posteriorly Displaced Distal Metaphyseal Clavicular Fracture (Type IV AC Joint Dislocation-Like) in Children: A Case Report and Literature Review Study

**DOI:** 10.1155/2016/4015212

**Published:** 2016-01-24

**Authors:** Ahmed Kotb, Taylor Yong, Amr Abdelgawad

**Affiliations:** ^1^School of Medicine, Ain Shams University, Cairo 11566, Egypt; ^2^Paul L. Foster School of Medicine, Texas Tech University Health Science Center, El Paso, TX 79905, USA

## Abstract

Fractures of the lateral end of the clavicle are common in pediatric patients; most of these fractures occur at the physeal level representing Salter Harris injuries. The vast majority of fractures of the lateral end of the clavicle are managed nonoperatively. In this report, we describe a unique type of fracture of the distal end of the clavicle in the pediatric patients in which the fracture occurs in the metaphyseal lateral clavicle with the proximal edge of the fracture displaced posteriorly through the trapezius muscle causing obvious deformity. It is similar in pathology to type IV AC joint dislocation. In this study we report this injury in eleven-year-old boy. Literature review showed that similar injuries were described before three times (two of them in pediatric patients). Due to the significant clinical deformity of this category with entrapment of the bone through the trapezius muscle, reduction (open or closed) of the fracture is the recommended treatment.

## 1. Background

The clavicle ranks among the most frequently fractured bones in the immature skeleton. It is prone to fracture as it resides subcutaneously along the majority of its length and distributes almost all forces from the upper extremity to the trunk. Fractures of the clavicle are categorized according to anatomic location, medial third, middle third, and distal third, the bulk of which involves the middle third, while distal fractures only represent 10–20% [1]. The distal clavicle is often injured following a direct blow to the AC joint [2].

As fusion of the distal epiphysis is not complete until the mid-twenties, fractures of the lateral end in children usually result in a physeal separation of the distal clavicle, rather than a true acromioclavicular (AC) separation [2–4]. Additionally, a thick periosteum forms a protective sleeve around the distal clavicle and the acromion and serves as a point of attachment for the coracoclavicular (CC) ligaments. Because the periosteum is weaker than the attachments of the ligaments, when fractures occur, the clavicle is displaced through a disruption in the periosteum rather than by detachment of the CC ligaments. Though these fractures may mimic AC separation (as the distal part of the fracture is still cartilaginous and radiolucent in children), they are more appropriately designated as “pseudodislocation” [4].

The purpose of this report is to describe a new category of distal clavicular fracture in children that occurs in the distal metaphysis and behaves like AC joint disruption type IV (posterior displacement). This category requires special attention as it needs reduction and not mere observation as in the majority of distal clavicular fractures. We present our case in addition to review of the literature for possible similar published cases. Pubmed search was used with cross reference of the articles. Studies describing distal clavicular fractures in children were assessed for possible similar cases.

## 2. Case Report

**Figure 1:**
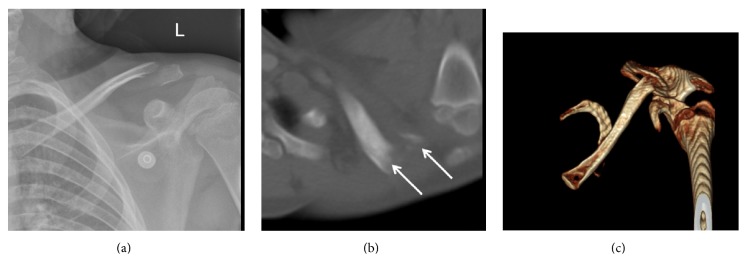
(a) Radiograph of the left shoulder showing fracture of the lateral metaphyseal clavicle. (b) CT scan of the left shoulder showing the posterior displacement of the proximal end of the fracture; arrows are pointing to the fracture ends. (c) 3D reformat of the CT of the left shoulder (view from above); notice the clavicle is posteriorly displaced in relation to the acromion and scapular spine.

**Figure 2:**
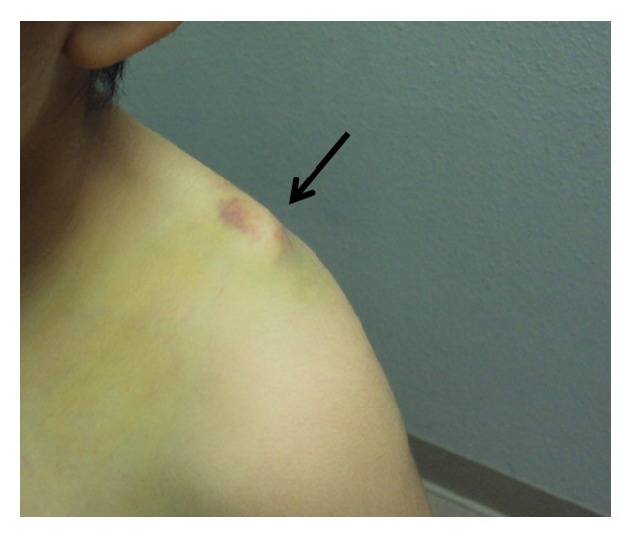
Clinical picture of the patient left shoulder showing the obvious deformity resulting from the posterior displacement of the fracture end (arrow).

An 11-year-old male was unconstrained in a motor vehicle collision. No loss of consciousness was reported. He presented to the emergency room with significant left shoulder pain. There was tenderness to palpation at the left shoulder and clavicle with minor overlying abrasion, erythema, and prominent posterior bony mass over his posterior shoulder. He was neurovascularly intact but was unable to move the shoulder due to significant pain. Radiography and CT revealed an oblique fracture of the distal third of the left clavicle with posterior displacement of the distal end of the proximal (medial) fragment (Figure 1). The fracture was in the metaphyseal portion of the distal clavicle. He was discharged from the emergency department with instructions to follow up with an orthopedic surgeon. He was subsequently evaluated in the orthopedic clinic. Because of the marked deformity of the posteriorly displaced fracture (Figure 2), an operative repair was advised, and the family agreed to proceed to surgery.

**Figure 3:**
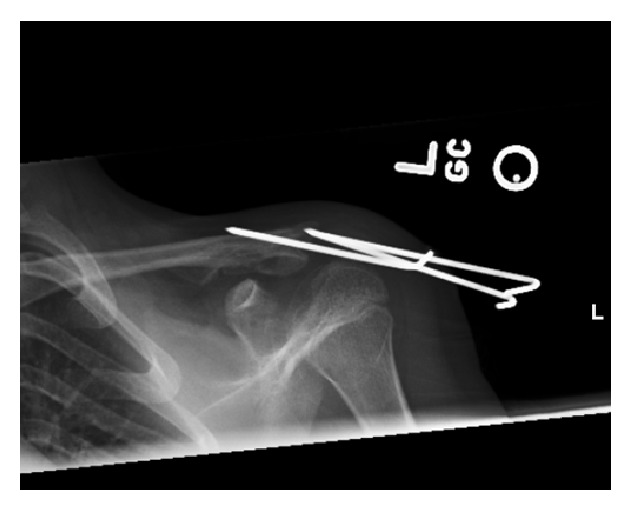
Radiographs of the left shoulder showing reduction of the clavicle fracture and fixation by three wires.

Patient underwent surgery one week following the initial injury. A small transverse incision was made over the distal clavicle. The periosteal sleeve around the distal end of the clavicle was disrupted; however, the attachment of the coracoclavicular ligament into the inferior part of the periosteal sleeve surrounding the clavicle was still intact. The distal end of the proximal (medial) fragment was observed to have disrupted the periosteal sleeve with significant posterior displacement into the trapezius muscle. Multiple attempts were required to free the distal end of the medial segment of the fracture from the trapezius muscle. Then, the fracture ends were reduced together. Once good reduction was achieved, three K-wires were inserted percutaneously from the lateral end of the acromion to the clavicle traversing both ends of the fracture (Figure 3). The patient's shoulder was immobilized in an arm sling. Four weeks after surgery, radiographs showed complete healing of the fracture in a good alignment. The wires were removed in the clinic and the patient was allowed to move his shoulder. At the time of final follow-up, eight weeks after surgery, patient had regained his full shoulder function and returned to full activity.

## 3. Discussion

Injuries involving the distal clavicle in children are classically “pseudodislocations” of the AC joint in which the joint and the coracoclavicular ligaments are usually intact, and fracture involves the lateral physis of the clavicle with displacement of the bone through a split in the periosteal sleeve. True dislocation typically does not occur because the AC joint is maintained by the trapezius and the deltoid muscles [1].

In the skeletally immature patient, ligamentous attachment by the coracoclavicular (CC) ligaments is biomechanically stronger than the physeal-metaphyseal region, thus allowing the lateral portion of the clavicle to be displaced from its periosteal tube, giving the radiographic impression of a dislocation of the AC joint due to radiolucent cartilaginous lateral physis.

The excellent remodeling capacity in immature bone allows most distal clavicular injuries to be treated nonoperatively. It is widely accepted that nondisplaced or minimally displaced fractures can be managed conservatively with sling immobilization and early rehabilitation with range-of-motion exercises [1, 5]. Kubiak and Slongo suggest that operative treatment is necessary only in older children or when the following indications exist: open fractures, impingement of soft tissue or potential risk for skin perforation, severe shortening of the shoulder girdle with or without displaced intermediate fragments, and displaced fractures with potential risk to the neurovascular bundle or mediastinal structures [6]. Another indication for surgical intervention involves the potential complication of formation of a bifid or Y-shaped clavicle which can cause discomfort and may require future reconstructive surgery [7].

Pediatric injuries that involve the AC joint have been classified by Dameron and Rockwood in a scheme that mirrors the classification of adult AC joint injuries. Type IV injury includes a posterior displacement of the distal clavicle in relation to the acromion with buttonholing of the shaft through the trapezius [8]. The injuries more commonly occur at the physeal region (pseudodislocation) or less commonly at the AC joint (true dislocation) [1]. The case presented here is clinically similar to type IV Dameron and Rockwood classification of AC injury in which there is a posterior displacement of the fracture end through the fibers of the trapezius muscle [9]. The difference between our case and true type IV AC dislocation is that in our case, the fracture was entirely through the metaphysis with no involvement of the joint or the physis of the distal clavicle as shown in the radiographs and CT cuts. However, we based our decision for open reduction and internal fixation of this fracture on the significant clinical deformity and on the current recommendations for operative treatment of types IV, V, and VI AC joint disruption due to the clinical similarity between our case and type IV AC joint disruption [5].

Literature review revealed three other similar cases with type IV-like fractures of the distal clavicle; two of these cases were in the pediatric age group [10, 11]. Itokazu et al. in 2001 reported a case involving an 11-year-old boy with a posteriorly displaced fracture of the distal clavicle and anchoring of the medial end of the clavicle into the trapezius muscle fibers that required surgical intervention [10]. A second case in the pediatric age group was reported by Richards and Howard involving a 13-year-old male with a fracture of the distal clavicle and a greater than 100 percent superior and posterior displacement of the clavicle with respect to the acromion. The patient underwent closed reduction under general anesthesia without the need for internal fixation [11]. The third case was presented by Goss and Li and involved a 63-year-old female patient with a significantly posteriorly displaced lateral clavicle fracture protruding through trapezius muscle. The segment was extricated from the muscle and reduced relative to the distal clavicular segment, and internal fixation was provided by 2 K-wires [12].

In conclusion, our case and the three other cases presented previously in the literature suggest that there is a separate category of distal clavicular fracture (more common in pediatric population) that involves the metaphysis of the distal clavicle with posterior displacement of the medial end of the proximal fracture segment through the trapezius. This fracture is similar to type IV AC joint injury, yet the fracture is entirely through the metaphysis of the distal clavicle. Due the significant clinical deformity of this category with entrapment of the bone through the trapezius muscle, reduction (open or closed) of the fracture is the recommended treatment.

## References

[1] Sarwark J. F., King E. C., Janicki J. A., Rockwood C. A., Wilkins K. E. (2010). Proximal humerus, scapula and clavicle. *Fractures in Children*.

[2] Bishop J. Y., Flatow E. L. (2005). Pediatric shoulder trauma. *Clinical Orthopaedics and Related Research*.

[3] Kirkos J. M., Papavasiliou K. A., Sarris I. K., Kapetanos G. A. (2007). A rare acromioclavicular joint injury in a twelve-year-old boy: a case report. *The Journal of Bone & Joint Surgery—American Volume*.

[4] Black G. B., McPherson J. A. M., Reed M. H. (1991). Traumatic pseudodislocation of the acromioclavicular joint in children. A fifteen year review. *The American Journal of Sports Medicine*.

[5] Abdelgawad A., Kanlic E., Abdelgawad A., Naga O. (2014). Orthopedic trauma. *Pediatric Orthopedics: Handbook for Primary Care Physicians*.

[6] Kubiak R., Slongo T. (2002). Operative treatment of clavicle fractures in children: a review of 21 years. *Journal of Pediatric Orthopaedics*.

[7] Ogden J. A. (1984). Distal clavicular physeal injury. *Clinical Orthopaedics and Related Research*.

[8] Barber F. A. (1987). Complete posterior acromioclavicular dislocation: a case report. *Orthopedics*.

[9] Nenopoulos S. P., Gigis I. P., Chytas A. A., Beslikas T. A., Nenopoulos A. S., Christoforidis J. E. (2011). Outcome of distal clavicular fracture separations and dislocations in immature skeleton. *Injury*.

[10] Itokazu M., Yoshida M., Itoh Y., Hukuta M., Kikuike K. (2001). Trapezius interposition of a distal third clavicular fracture in a child: a case report. *Journal of Orthopaedic Surgery*.

[11] Richards D. P., Howard A. (2001). Distal clavicle fracture mimicking type IV acromioclavicular joint injury in the skeletally immature athlete. *Clinical Journal of Sport Medicine*.

[12] Goss T. P., Li X. (2012). A variant of a type V lateral clavicle fracture involving a posteriorly displaced medial segment. A case report. *Sports Medicine, Arthroscopy, Rehabilitation, Therapy & Technology*.

